# A stage-based dynamic model for predicting bronchopulmonary dysplasia in very preterm infants using longitudinal lung ultrasound: a retrospective cohort study

**DOI:** 10.3389/fped.2026.1928918

**Published:** 2026-10-28

**Authors:** Huihui Yang, Xiongpeng He, Xijie Xu, Xingpei Li, Jianyi Liao, Yu He

**Affiliations:** Department of Ultrasound Medicine, Guangdong Provincial Key Laboratory of Major Obstetric Diseases, Guangdong Provincial Clinical Research Center for Obstetrics and Gynecology, The Third Affiliated Hospital, Guangzhou Medical University, Guangzhou, China

**Keywords:** dynamic prediction model, bronchopulmonary dysplasia, very preterm infants, lung ultrasound, lung ultrasound score

## Abstract

**Background:**

Bronchopulmonary dysplasia (BPD) remains a major complication in very preterm infants (VPIs), and static clinical models may not capture evolving neonatal lung injury. We assessed whether a stage-based model using serial lung ultrasound (LUS) could improve early BPD prediction.

**Methods:**

In this retrospective single-center cohort, VPIs born at <32 weeks' gestation between January 2021 and December 2022 underwent LUS on postnatal days 7, 14, 21, and 28. Early clinical variables were collected, and BPD was defined by the 2018 NICHD criteria at 36 weeks’ postmenstrual age. Single-time-point models were compared with stage-based dynamic models incorporating prior predicted risk and later LUS data, using bootstrap out-of-bag validation.

**Results:**

Among 269 VPIs, 174 developed BPD. LUS scores were higher in infants who developed BPD at all assessed time points. The best-performing single-time-point model used day-14 LUS (AUC 0.853, 95% CI 0.811–0.892). The three-stage random forest model achieved the highest point-estimate discrimination (AUC 0.874, 95% CI 0.835–0.909). Compared with the best-performing single-time-point model, the three-stage strategy showed an improvement in AUC of +0.021 (95% CI +0.005-+0.038). Compared with the two-stage strategy, the incremental difference in AUC was +0.009 (95% CI +0.001 to +0.018), whereas extension to the four-stage strategy showed no clear additional improvement (ΔAUC 0.006, 95% CI−0.004 to +0.016). In the three-stage model, prior-stage predicted risk and day-21 LUS remained independently associated with BPD.

**Conclusions:**

Serial LUS-based risk updating provided incremental prognostic information beyond single-time-point assessment. The three-stage model incorporating information through postnatal day 21 achieved the highest point-estimate discrimination, whereas extension to day 28 provided no clear additional predictive gain.

## Introduction

Bronchopulmonary dysplasia (BPD) remains one of the most common and serious chronic pulmonary complications in very preterm infants (VPIs) (1), defined as those born before 32 weeks of gestation (2). Despite substantial improvements in perinatal care and neonatal intensive care management, the incidence of BPD has not decreased accordingly (1, 3). Infants with BPD often experience prolonged hospitalization, recurrent respiratory infections, impaired lung function, and adverse neurodevelopmental outcomes, imposing a substantial medical and socioeconomic burden (4–6). Therefore, early identification of infants at high risk for BPD and timely intervention are critical for improving clinical outcomes.

The development of BPD is multifactorial, involving complex interactions between pulmonary immaturity, oxygen toxicity, mechanical ventilation, inflammation, and impaired vascular and alveolar development (7–9). Although previous studies have proposed various predictive models based on perinatal or early postnatal clinical characteristics, most rely on static variables such as gestational age, birth weight, or respiratory support parameters (10, 11). These indicators fail to capture the dynamic nature of lung injury and recovery in the early neonatal period (12). Moreover, existing models often lack external validation and show inconsistent predictive accuracy across different populations, limiting their clinical utility.

Lung ultrasound (LUS) has emerged as a valuable, noninvasive, and radiation-free imaging technique for assessing pulmonary aeration and interstitial changes in preterm infants (9, 13). Compared with traditional radiography, LUS allows real-time and repeated bedside evaluations, providing dynamic insights into lung conditions (14, 15). Several studies have shown that LUS scores correlate with the subsequent development of BPD; however, most research has been limited to single-time-point measurements obtained during the first postnatal weeks (16, 17). Considering that pulmonary disease in VPIs evolves rapidly, a dynamic, multi-time-point evaluation of LUS may better capture the temporal trajectory of lung pathology and improve predictive performance (18).

Based on this rationale, this retrospective single-center study analyzed clinical and imaging data of very preterm infants to explore the predictive value of multi-time-point LUS assessments combined with clinical features for the early identification of BPD. Multivariate logistic regression analysis was used to identify independent risk factors, and the predictive performance was evaluated at different stages to determine the optimal predictive structure.

Although single-time-point LUS already achieves moderate discrimination (pooled AUC 0.85–0.87 in meta-analyses), it remains unknown whether stage-based dynamic updating adds incremental prognostic value beyond a single early assessment. The novelty of this study therefore lies not in the absolute performance of LUS at any single time point, but in (1) extending LUS assessment beyond an isolated early measurement to evaluate the incremental value of serial assessments across the postnatal period, and (2) iteratively updating BPD risk using longitudinal LUS information within a stage-based framework.

## Methods

### Study design

This was a single-center retrospective cohort study conducted in the neonatal intensive care unit (*N*ICU) of the third affiliated hospital of Guangzhou Medical University. The study was reported in accordance with the TRIPOD (Transparent Reporting of a multivariable prediction model for Individual Prognosis Or Diagnosis) statement (19). Very preterm infants admitted between January 2021 and December 2022 were screened for eligibility. The primary objective was to develop and compare dynamic prediction models for BPD based on lung ultrasound (LUS) findings obtained at multiple postnatal time points in combination with clinical variables, and to evaluate the predictive performance of different time windows and stage-based modeling strategies.

### Study population

Very preterm infants were defined as infants born at <32 weeks’ gestational age (GA). Infants were eligible for inclusion if they met all of the following criteria:(1) GA < 32 weeks; (2) admission to the NICU within 24 h after birth; (3) complete perinatal and in-hospital clinical data; and (4) completion of standardized lung ultrasound examinations on postnatal days 7, 14, 21, and 28. Completion of all four serial LUS examinations was required for inclusion to ensure complete longitudinal data for stage-based modeling. Infants were excluded if they met any of the following criteria: (1) death, transfer to another hospital, or withdrawal of care before 36 weeks’ postmenstrual age (PMA); (2) presence of major congenital malformations, chromosomal abnormalities, or inherited metabolic disorders; or (3) missing key clinical or imaging data precluding model construction. According to the occurrence of BPD, infants were categorized into the BPD group and the non-BPD group.

### Ethical considerations

This study was approved by the Institutional Ethics Committee of the Third Affiliated Hospital of Guangzhou Medical University (Approval No：2024211). Written broad consent was obtained from parents or legal guardians before clinical examinations, permitting de-identified examination results to be used for scientific research. For infants whose parents or legal guardians had not provided broad consent, verbal informed consent was obtained by telephone. For the small number of families who could not be reached by telephone after reasonable attempts, a waiver of informed consent was granted by the ethics committee, and only de-identified examination results were used for this study. The study was conducted in accordance with the Declaration of Helsinki.

### Clinical variables and outcome definition

Demographic and perinatal variables collected included sex, gestational age, birth weight, Apgar scores, mode of delivery, premature rupture of membranes, antenatal corticosteroid exposure, and surfactant administration. In-hospital clinical variables included type and duration of respiratory support, fraction of inspired oxygen (FiO₂), invasive mechanical ventilation, anemia, infection, and the presence and treatment of hemodynamically significant patent ductus arteriosus (hsPDA); hsPDA was diagnosed by echocardiography performed when clinically indicated, as documented in the medical records. These variables were collected for cohort characterization and as candidate predictors; detailed phenotypic analyses stratified by respiratory support pattern were not part of the prespecified analysis plan.

In-hospital variables were extracted for cohort characterization. Only variables determined antenatally or available within the first 7 postnatal days were eligible as candidate predictors for model development; variables occurring later during hospitalization were used for descriptive purposes only and were not entered into the prediction models.

The primary outcome was BPD defined according to the 2018 NICHD consensus criteria, based on the level of respiratory support at 36 weeks’ postmenstrual age (20).

### Lung ultrasound examination and scoring

Lung ultrasound examinations were performed on postnatal days 7, 14, 21, and 28 while infants were in a calm state or natural sleep. Bedside lung ultrasound was conducted using a Philips CX50 ultrasound system (Philips Healthcare) equipped with a linear-array transducer (L12-3) operating at 8–12 MHz. Examinations were conducted following a standardized neonatal lung ultrasound protocol recommended by the Chinese neonatal lung ultrasound diagnostic guideline (21). Both lungs were divided into 12 regions, with each lung separated into anterior, lateral, and posterior zones by the anterior and posterior axillary lines; each zone was further subdivided into upper and lower regions, yielding six regions per lung (12 regions in total).

Each region was scored using a four-level semi-quantitative aeration score (0–3), with scoring principles aligned with the neonatal lung ultrasound scoring semiology described by Brat et al. (22): score 0, normal lung pattern with A-lines; score 1, interstitial syndrome with the presence of B-lines; score 2, severe interstitial syndrome with coalescent B-lines; and score 3 (Figure 1), extended lung consolidation. This semiology-based grading reflects progressive loss of lung aeration and is consistent with internationally accepted lung ultrasound terminology. The highest score observed in each region was recorded, and the total lung ultrasound score was calculated as the sum of all regional scores, ranging from 0 to 36, with higher scores indicating more severe loss of lung aeration.

**Figure 1:**
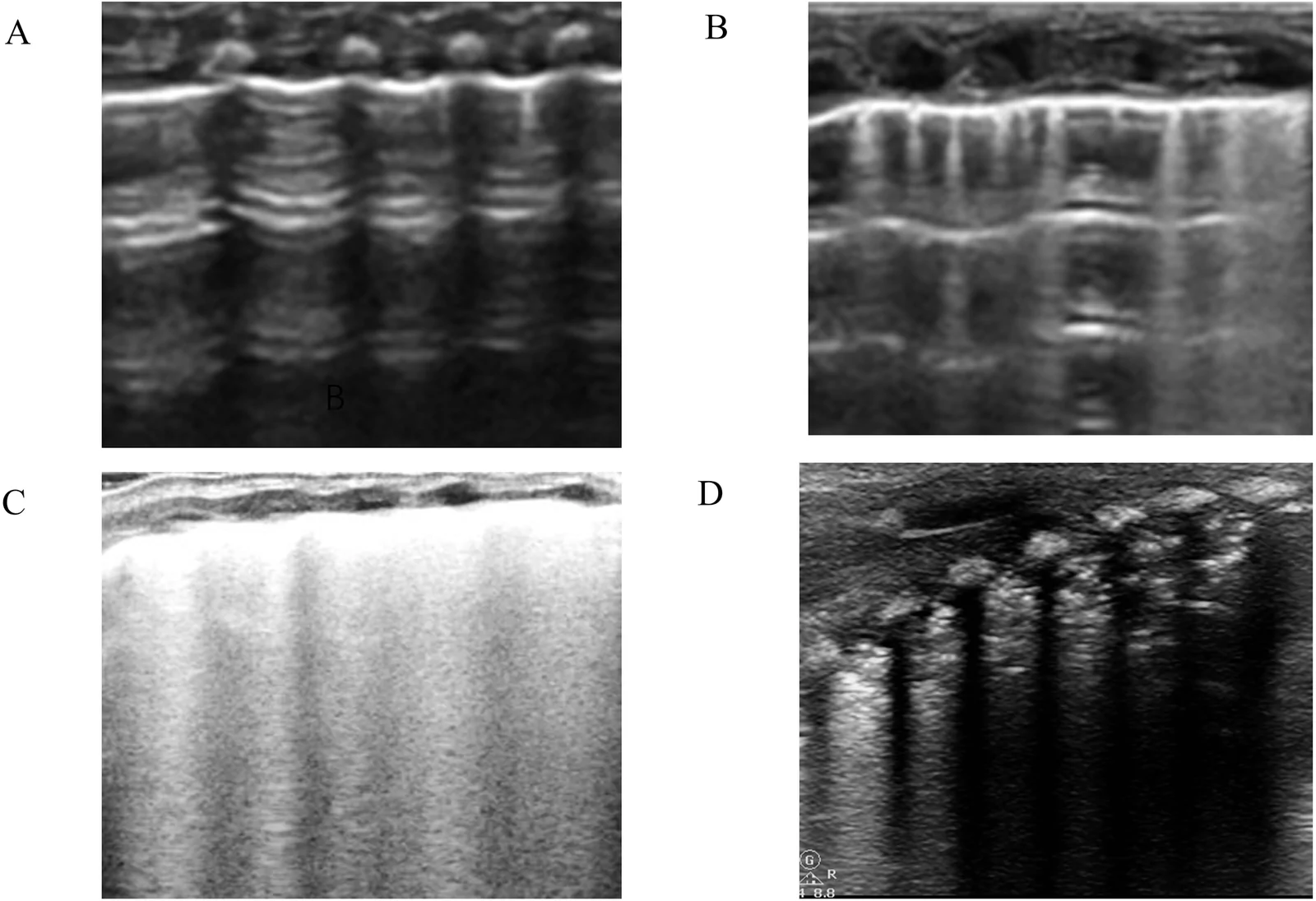
Representative lung ultrasound images illustrating the four-level semi-quantitative aeration score (0–3) used in this study. **(A)** Score 0: normal lung pattern with horizontal A-lines. **(B)** Score 1: interstitial syndrome with isolated vertical B-lines. **(C)** Score 2: severe interstitial syndrome with coalescent B-lines. **(D)** Score 3: extended lung consolidation with air bronchograms.

All ultrasound images were independently scored by three physicians experienced in neonatal lung ultrasound who were blinded to clinical outcomes, and the mean score was used for analysis. When discrepancies between scorers exceeded one point in any lung region, the images were reviewed by an additional senior expert for adjudication and a consensus score was reached.

### Data collection

All demographic, perinatal, and in-hospital clinical data were extracted from the electronic medical record system by two investigators independently and cross-checked for accuracy. Lung ultrasound images were acquired using a uniform protocol, centrally stored, and reviewed for scoring and analysis. Data completeness, consistency, and plausibility were verified prior to model development.

### Model development and statistical analysis

All statistical analyses were performed using Python (version 3.9.18; Python Software Foundation, Wilmington, DE, USA). Continuous variables were assessed for normality and summarized as mean ± standard deviation or median (interquartile range), as appropriate. Categorical variables were summarized as counts and percentages. Group comparisons were conducted using the independent-samples *t*-test or Mann–Whitney *U*-test for continuous variables and the chi-square test for categorical variables.

BPD occurrence was used as the dependent variable. Candidate predictors included baseline clinical variables and LUS scores. For feature selection, an L1-penalized logistic regression (LASSO-type) approach was applied on the training data within each resampling iteration to retain predictors with non-zero coefficients. Multivariable logistic regression was used to develop interpretable prediction models, and additional machine learning algorithms were evaluated to capture potential nonlinear relationships, including random forest (RF), histogram-based gradient boosting trees (HGBT), multilayer perceptron (MLP), and a stacking ensemble model. All machine learning models were implemented using scikit-learn.

Based on the availability of LUS data at different postnatal time points, the following prediction strategies were constructed: (1) single-stage models incorporating baseline clinical variables and the LUS score from a single postnatal time point; and (2) stage-based dynamic models, in which predicted risk from the previous stage was generated within the training data (using internal cross-validation) and then included as an input feature for subsequent stages, allowing construction of two-stage, three-stage, and four-stage models.

Model performance was evaluated using a bootstrap-out-of-bag (OOB) internal validation framework with 1,000 bootstrap resamples. In each resample, the full modeling pipeline (previous-stage risk generation, feature selection, and model fitting) was performed on the bootstrap sample, and probabilistic predictions were obtained only for the corresponding OOB observations. For each infant, OOB predicted probabilities were aggregated across resamples, and the reported point estimates of model performance were calculated from these aggregated OOB predictions. To quantify uncertainty, AUC and Brier score were additionally calculated within each resample-specific OOB set, and 95% CIs were obtained from the 2.5th and 97.5th percentiles of the corresponding bootstrap distributions. For comparisons between staging strategies, differences in AUC and Brier score were calculated within the same bootstrap resample, thereby preserving the paired structure of the predictions, and percentile-based 95% CIs were derived from the resulting paired difference distributions. Model calibration was evaluated using calibration plots and the Hosmer-Lemeshow test. Clinical utility was assessed using decision curve analysis (DCA). All statistical tests were two-sided, and a *P* < 0.05 was considered statistically significant.

## Results

### Study cohort

The process of cohort derivation is shown in Figure 2. A total of 457 preterm infants were screened during the study period. Of these, 121 infants with a gestational age ≥32 weeks were excluded, leaving 336 confirmed very preterm infants (GA < 32 weeks). A further 13 infants who were admitted to the NICU more than 24 h after birth were excluded, and 323 infants proceeded to full eligibility assessment. Among these, 54 infants were excluded according to the prespecified exclusion criteria: 28 because of death, transfer, or withdrawal of care before 36 weeks’ postmenstrual age, 14 because of major congenital anomalies, chromosomal abnormalities, or inherited metabolic disorders, and 12 because of missing key clinical or lung ultrasound data. The final analytic cohort therefore consisted of 269 infants, including 174 with bronchopulmonary dysplasia (BPD) and 95 without BPD.

**Figure 2:**
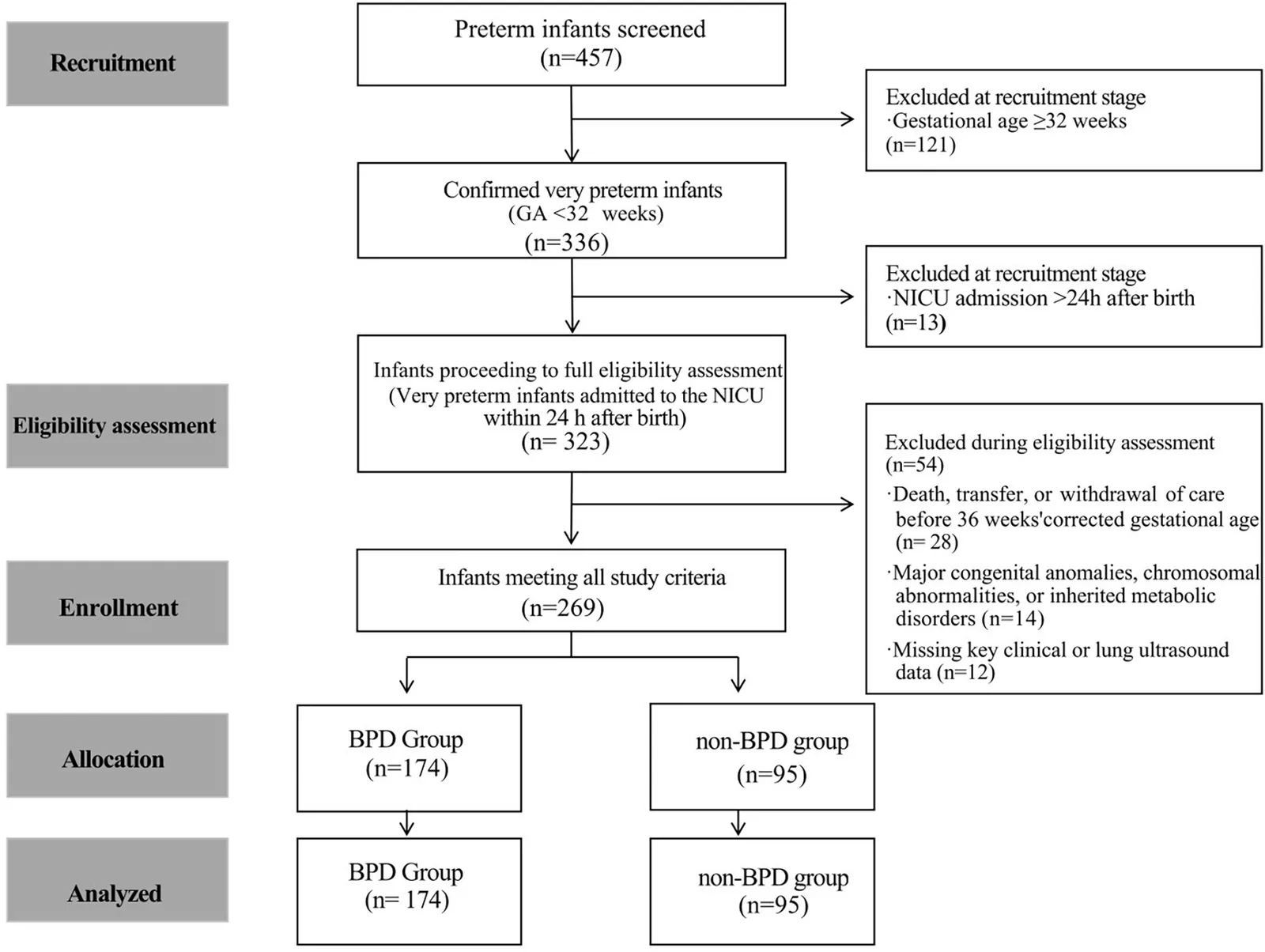
Flow diagram of participant recruitment, eligibility assessment, enrollment, and group allocation. A total of 457 preterm infants were initially screened. After excluding 121 infants with a gestational age ≥32 weeks, 336 were confirmed as very preterm infants (GA < 32 weeks). Thirteen infants admitted to the NICU more than 24 h after birth were subsequently excluded, leaving 323 infants for full eligibility assessment. A further 54 infants were excluded according to the prespecified exclusion criteria, resulting in a final analytic cohort of 269 infants, including 174 with BPD and 95 without BPD.

### Baseline characteristics

Baseline neonatal, maternal, and perinatal characteristics are summarized in Table 1 and Supplementary Table S1. As expected, infants who developed BPD were born more prematurely and with lower birth weight than those who did not. Median gestational age was 28.00 weeks in the BPD group compared with 30.00 weeks in the non-BPD group, and mean birth weight differed by more than 250 g between groups (both *P* < 0.001).

**Table 1:** Baseline characteristics of infants with and without bronchopulmonary dysplasia.

Variable	Non-BPD group (*n* = 95)	BPD group (*n* = 174)	*P* value
Neonatal characteristics			
Gestational age (weeks)	30.00 [29.36, 30.86]	28.00 [26.71, 29.57]	<0.001*
Sex			0.299
Male	50/95 (52.6%)	103/174 (59.2%)	
Female	45/95 (47.4%)	71/174 (40.8%)	
Birth weight (g)	1,283.00 ± 309.29	1,027.49 ± 284.96	<0.001*
Birth asphyxia			0.151
None	60/95 (63.2%)	102/174 (58.6%)	
Mild	27/95 (28.4%)	65/174 (37.4%)	
Severe	8/95 (8.4%)	7/174 (4.0%)	
Apgar score			
1-min Apgar score	8.00 [6.00, 10.00]	8.00 [7.00, 9.00]	0.243
5-min Apgar score	10.00 [9.00, 10.00]	9.00 [9.00, 10.00]	0.259
10-min Apgar score	10.00 [9.00, 10.00]	10.00 [9.00, 10.00]	0.671
LUS scores and related interventions			
LUS at day 7	12.00 [10.00, 13.00]	18.00 [13.00, 22.00]	<0.001*
LUS at day 14	12.00 [9.50, 14.00]	18.00 [15.00, 24.00]	<0.001*
LUS at day 21	12.00 [11.00, 14.00]	18.00 [14.00, 23.75]	<0.001*
LUS at day 28	12.00 [10.00, 12.00]	16.00 [13.00, 21.00]	<0.001*
Time from birth to surfactant administration (min)	67.71 [10.00, 170.83]	15.00 [10.00, 85.16]	0.001*
Surfactant administration mode			<0.001*
None	14/95 (14.7%)	15/174 (8.6%)	
MIST	4/95 (4.2%)	4/174 (2.3%)	
INSURE	58/95 (61.1%)	47/174 (27.0%)	
Intubation	19/95 (20.0%)	108/174 (62.1%)	
Respiratory support			
Mechanical ventilation within 72 h			<0.001*
No	80/95 (84.2%)	91/174 (52.3%)	
Yes	15/95 (15.8%)	83/174 (47.7%)	
Maternal and perinatal characteristics			
Maternal age (years)	32.71 ± 4.40	31.79 ± 5.36	0.131
Pre-pregnancy weight (kg)	54.00 [48.00, 60.00]	55.00 [50.00, 60.75]	0.173
Gravidity	2.00 [1.00, 3.00]	2.00 [1.00, 3.00]	0.502
Parity	1.00 [1.00, 2.00]	1.00 [1.00, 2.00]	0.965
Intrauterine distress			0.195
No	80/95 (84.2%)	135/174 (77.6%)	
Yes	15/95 (15.8%)	39/174 (22.4%)	
Amniotic fluid abnormality			0.006*
Normal	83/95 (87.4%)	133/174 (76.4%)	
Abnormal	12/95 (12.6%)	41/174 (23.5%)	

BPD, bronchopulmonary dysplasia; LUS, lung ultrasound; MIST, minimally invasive surfactant therapy; INSURE, intubation–surfactant–extubation.

* *P* < 0.05 indicates statistical significance.

Several early clinical features also differed between groups. Lung ultrasound (LUS) scores were consistently higher in infants who later developed BPD at all assessed postnatal time points (days 7, 14, 21, and 28), with significant between-group differences at each time point (all *P* < 0.001). Differences in early respiratory management were evident as well. Infants in the BPD group received surfactant earlier after birth, and intubation-based surfactant administration was substantially more common. In addition, mechanical ventilation within the first 72 h of life occurred substantially more frequently in infants who developed BPD (47.7% vs. 15.8%).

By contrast, several baseline characteristics showed no meaningful between-group differences. Sex distribution, severity of birth asphyxia, and Apgar scores at 1, 5, and 10 min were comparable between groups. Among maternal and perinatal factors, amniotic fluid abnormality and maternal anemia were more frequent in the BPD group, whereas most other obstetric and antenatal variables did not differ significantly.

### Performance of single-time-point prediction models

The discriminative performance of single-time-point models based on LUS assessments is shown in Figure 3 and Supplementary Table S2. Across postnatal days 7, 14, 21, and 28 and across all algorithms, out-of-bag (OOB) AUC values ranged from approximately 0.80–0.85. Among these strategies, models incorporating LUS measurements obtained at postnatal day 14 consistently achieved the highest discrimination, with AUC values reaching 0.853(95% CI: 0.811–0.892).

**Figure 3:**
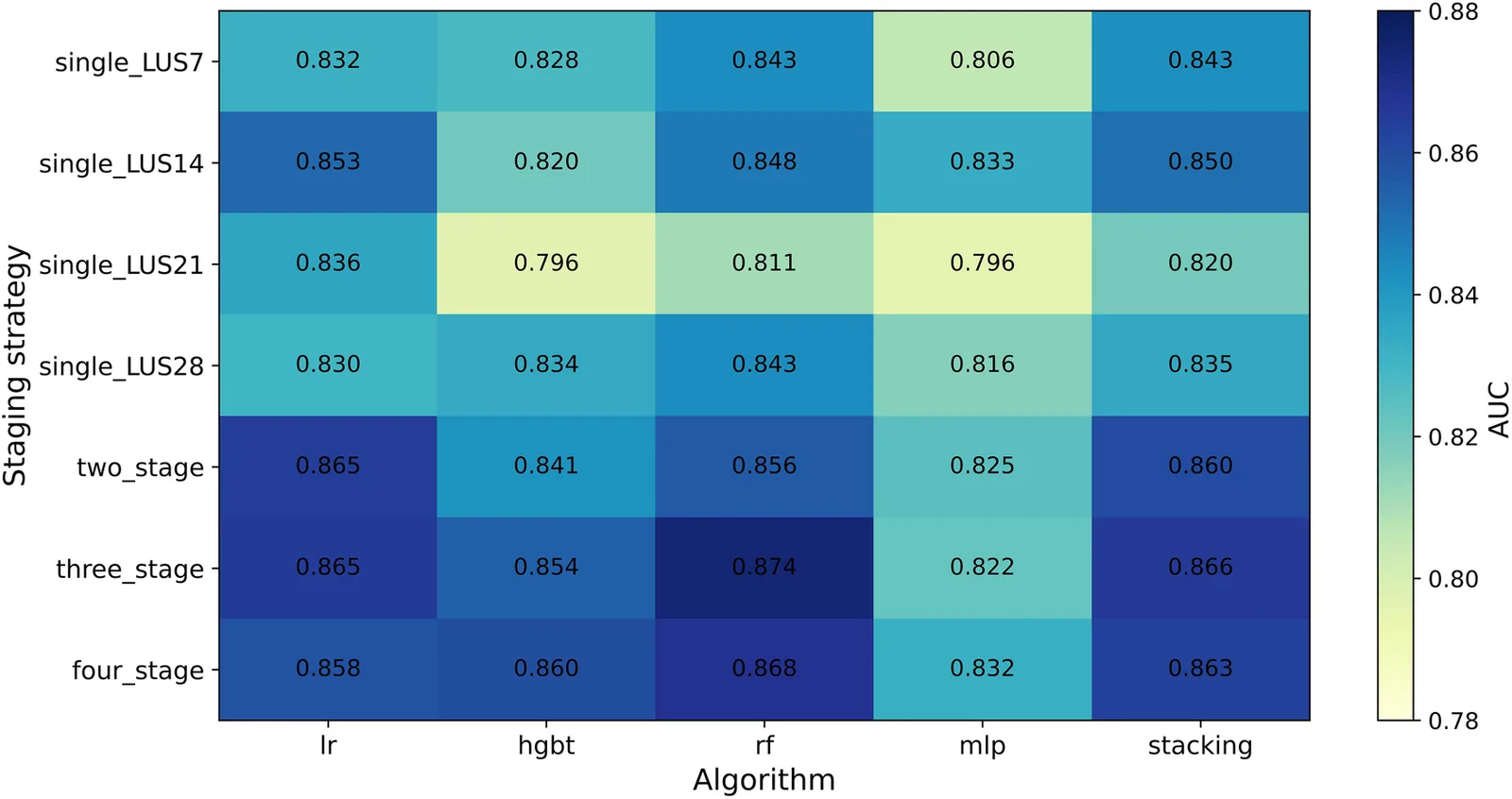
Heatmap of predictive performance for single-time-point and multi-time-point models across different algorithms. The heatmap illustrates the area under the receiver operating characteristic curve (AUC) for predictive models developed using different algorithms and lung ultrasound–based staging strategies. Columns represent the machine learning algorithms, including logistic regression (LR), histogram-based gradient boosting trees (HGBT), random forest (RF), multilayer perceptron (MLP), and stacking ensemble. Rows represent the staging strategies, including single-time-point models at different postnatal time points (single_LUS7, single_LUS14, single_LUS21, and single_LUS28) and multi-time-point models (two-stage, three-stage, and four-stage). Each cell shows the out-of-bag AUC value, with darker colors indicating higher discriminative performance. Overall, multi-time-point strategies generally showed higher point-estimate AUCs than single-time-point strategies across the evaluated algorithms, although this pattern was not uniform across all algorithms. The three-stage strategy achieved the highest overall point-estimate AUC among the selected best-performing models, whereas extension to the four-stage strategy produced comparable performance. Formal paired comparisons and their 95% CIs are reported in Supplementary Table S5.

Classification performance varied by algorithm and time point. Sensitivity ranged from moderate to high, whereas specificity generally remained above 0.70. Positive and negative predictive values for all single-time-point models are detailed in Supplementary Table S2.

### Performance of multi-stage prediction models

Predictive performance of multi-stage models is summarized in Figure 3 and Table 2. Multi-stage strategies generally yielded higher point-estimate AUCs than single-time-point strategies, although this pattern was not uniform across all algorithms.

**Table 2:** Out-of-bag predictive performance of single-time-point and multi-stage models across different staging strategies.

Staging strategy	Best algorithm	AUC (OOB), 95% CI	Brier score (OOB), 95% CI
Single_LUS7	RF	0.843 (0.797–0.885)	0.158 (0.139–0.178)
Single_LUS14	LR	0.853 (0.811–0.892)	0.148 (0.130–0.168)
Single_LUS21	LR	0.836 (0.789–0.879)	0.157 (0.138–0.177)
Single_LUS28	RF	0.843 (0.797–0.884)	0.161 (0.141–0.182)
two_stage	LR	0.865 (0.824–0.901)	0.140 (0.123–0.158)
three_stage	RF	0.874 (0.835–0.909)	0.137 (0.121–0.155)
four_stage	RF	0.868 (0.827–0.904)	0.137 (0.121–0.154)

OOB, out-of-bag; OOF, out-of-fold; AUC, area under the receiver operating characteristic curve; LR, logistic regression; RF, random forest; LUS, lung ultrasound.

**Model definitions:** Single_LUS7, Single_LUS14, Single_LUS21, and Single_LUS28 represent single-time-point models constructed using baseline clinical variables together with the lung ultrasound (LUS) score obtained at postnatal day 7, 14, 21, or 28, respectively.

Multi-time-point models were developed in a stage-based manner. The two-stage model integrates LUS information from postnatal days 7 and 14; the three-stage model integrates LUS information from postnatal days 7, 14, and 21; and the four-stage model integrates LUS information from postnatal days 7, 14, 21, and 28. At each stage, a predictive model is trained using baseline variables and the current LUS measurement, and the out-of-fold predicted BPD risk from the preceding stage is incorporated as an input feature for the subsequent stage.

Two-stage models achieved OOB AUC values between 0.841 and 0.865, with the logistic regression–based model performing best among them. The two-stage logistic regression model achieved an OOB AUC of 0.865 (95% CI 0.824–0.901) and a Brier score of 0.140 (95% CI 0.123–0.158). The three-stage random forest model achieved the highest point-estimate AUC among the evaluated strategies (0.874, 95% CI0.835–0.909), with a Brier score of 0.137 (95% CI 0.121–0.155).

Compared with the best-performing single-time-point model at postnatal day 14, the three-stage strategy showed a ΔAUC of 0.021 (95% CI 0.005–0.038). Compared with the two-stage strategy, the corresponding differences were 0.009 (95% CI 0.001–0.018) for AUC. The difference between the three- and four-stage strategies was small (ΔAUC 0.006, 95% CI −0.004 to +0.016), indicating no clear additional predictive benefit from incorporating the day-28 assessment. Paired comparisons are summarized in Supplementary Table S5.

The robustness of these findings was further confirmed in sensitivity analyses using alternative model specifications, which showed highly consistent performance across different modeling approaches (Supplementary Table S3).

Sensitivity and specificity estimates derived from confusion matrices are provided in Supplementary Table S2. For three-stage models, sensitivity ranged from 0.75 to 0.83 and specificity from 0.80 to 0.85 across algorithms, indicating a balanced trade-off between sensitivity and specificity.

### ROC characteristics and clinical utility

Receiver operating characteristic curves for the best-performing algorithm at each staging strategy are shown in Figure 4. Multi-stage strategies generally showed higher point-estimate discrimination than single-time-point strategies. The three-stage model achieved the highest point-estimate AUC among the evaluated strategies, while the magnitude and uncertainty of between-strategy differences are reported in Supplementary Table S5.

**Figure 4:**
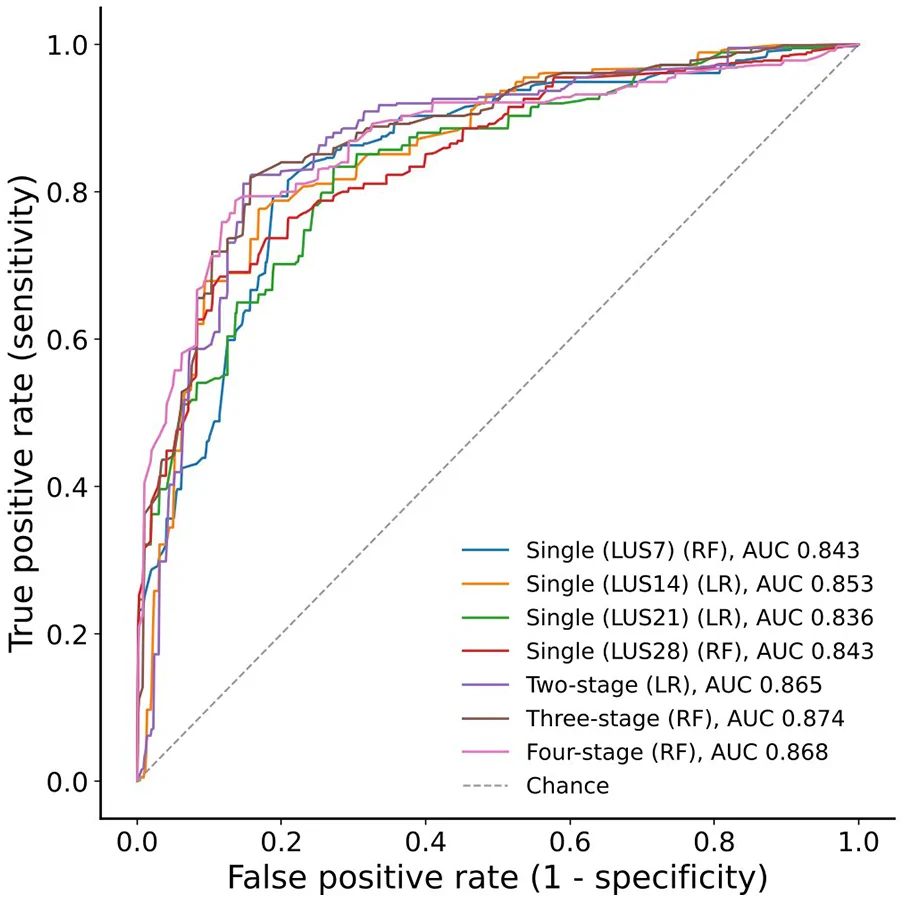
Receiver operating characteristic curves of the best-performing algorithms for each staging strategy. Receiver operating characteristic (ROC) curves are shown for the best-performing algorithm under each staging strategy. For each single-time-point model (single_LUS7, single_LUS14, single_LUS21, and single_LUS28) and multi-time-point model (two-stage, three-stage, and four-stage), the algorithm with the highest AUC calculated from aggregated out-of-bag (OOB) predicted probabilities was selected. ROC curves were generated using the corresponding aggregated OOB predicted probabilities, and the AUC values are reported in the figure legend. The diagonal dashed line represents the performance of a non-informative classifier.

Clinical utility was further assessed using decision curve analysis and risk stratification (Figure 5). Across a wide range of threshold probabilities, multi-stage models provided greater net benefit than default treat-all or treat-none strategies. This benefit was consistently observed for the three-stage model. Risk stratification analyses revealed clear gradients in observed BPD incidence. In the three-stage model, observed BPD rates increased from 0.26 in the low-risk group to 0.74 in the medium-risk group and 0.94 in the high-risk group, indicating effective separation of clinically meaningful risk categories.

**Figure 5:**
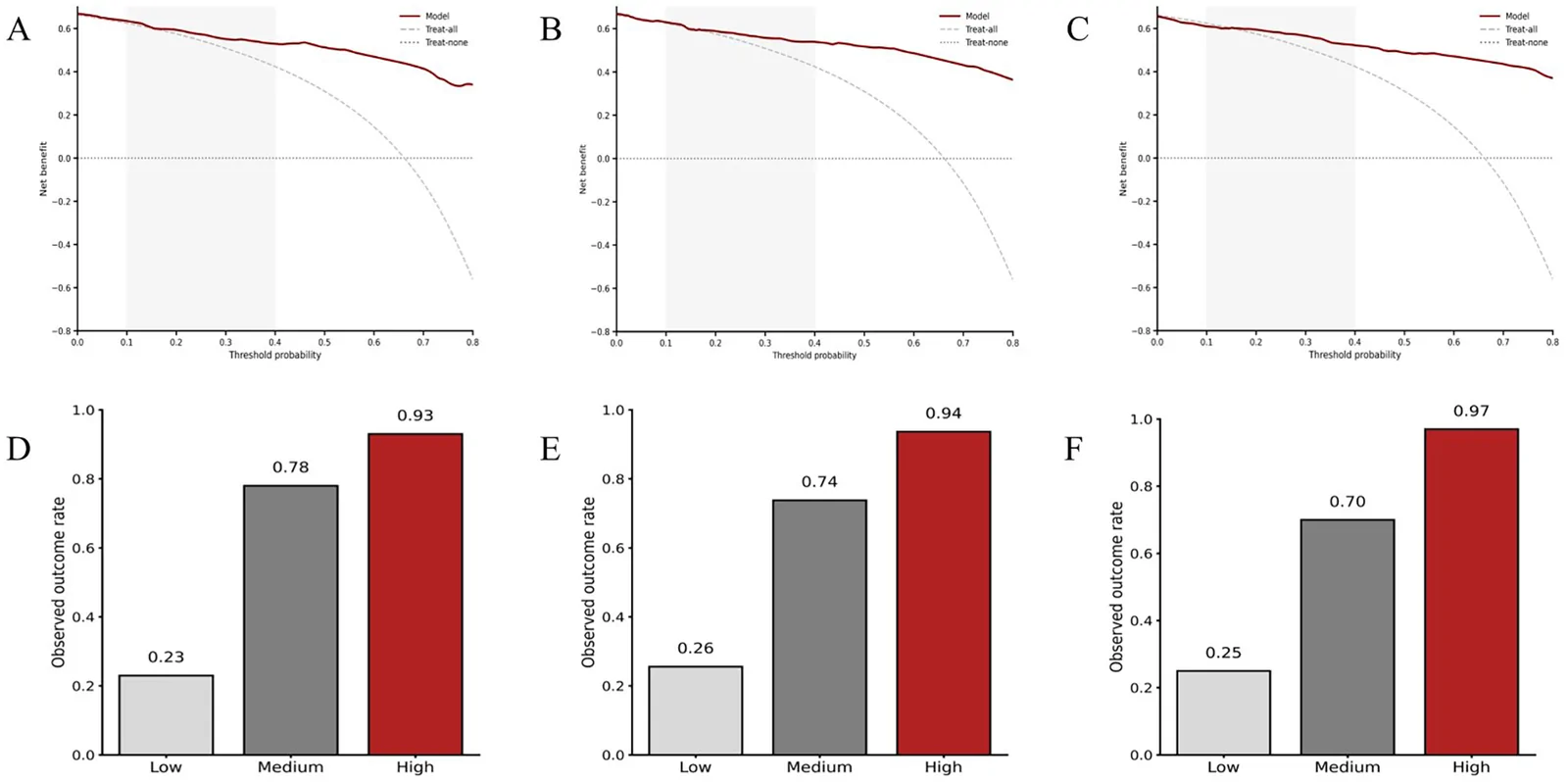
Clinical utility and risk stratification performance of multi-time-point prediction models. Panels **(A–C)** show decision curve analysis (DCA) for the two-stage **(A)**, three-stage **(B)**, and four-stage **(C)** models, respectively. The net benefit of each model across a range of threshold probabilities is compared with default strategies of treating all patients or treating none. Panels **(D–F)** present risk stratification analyses corresponding to the two-stage **(D)**, three-stage **(E)**, and four-stage **(F)** models. Patients were stratified into low-, medium-, and high-risk groups based on predicted probabilities, and the observed outcome rates are shown for each risk category. Together, these analyses demonstrate the incremental clinical benefit and improved risk discrimination achieved by multi-time-point models, particularly for the three-stage and four-stage approaches.

### Stage-based multivariable associations and feature importance

Multivariable logistic regression results for the three-stage model are presented in Table 3. After LASSO selection, 13 predictors were retained for model construction. Multivariable logistic regression analysis showed that the out-of-fold predicted risk from the preceding day-14 stage (_prev14) and the contemporaneous LUS score at postnatal day 21 remained independently associated with BPD. None of the baseline clinical variables demonstrated independent associations after adjustment, and variance inflation factor analysis did not suggest problematic multicollinearity.

**Table 3:** Multivariable logistic regression analysis of the three-stage model.

**Variable**	**OR**	**95% CI**	***P* value**
Prior-stage risk information			
OOF-predicted BPD risk from day-14 stage (_prev14)	**3**.**09**	**2.08**–**4.58**	**<0**.**001**
Current-stage LUS variable			
LUS score at postnatal day 21 (LUS21)	**3**.**44**	**1.85**–**6.40**	**<0**.**001**
Baseline clinical variables			
Pre-pregnancy weight	1.08	0.72–1.64	0.698
Gravidity	0.97	0.63–1.49	0.883
Parity	0.88	0.58–1.34	0.542
Maternal age	0.85	0.58–1.24	0.394
Neonatal Sex	1.05	0.72–1.52	0.798
PROM duration (h)	0.94	0.67–1.32	0.714
Time since last antenatal steroid dose (h)	1.11	0.80–1.54	0.534
Time from birth to surfactant administration (min)	0.92	0.64–1.35	0.683
Apgar score (1 min)	1.21	0.79–1.86	0.382
Apgar score (5 min)	1.07	0.57–2.01	0.830
Apgar score (10 min)	0.83	0.29–2.33	0.722

BPD, bronchopulmonary dysplasia; OR, odds ratio; CI, confidence interval; LUS, lung ultrasound; PROM, premature rupture of membranes; OOF, out-of-fold.

Variance inflation factor analysis did not indicate concerning multicollinearity among the included variables.

Model definitions: The three-stage model integrates lung ultrasound (LUS) scores obtained at postnatal days 7, 14, and 21 in a stage-based manner. At each stage, a predictive model is trained using baseline clinical variables and the current LUS measurement. The out-of-fold predicted probability of BPD from the preceding stage (_prev14) is incorporated as an input feature at the subsequent stage.

Bold values indicate statistically significant associations (*P* < 0.05).

Feature importance analyses across different algorithms showed a highly consistent pattern (Supplementary Table S4). In all three-stage models, the prior-stage predicted risk (_prev14) was the dominant contributor to prediction, followed by the current LUS measurement. Baseline clinical variables contributed modestly and variably depending on the algorithm. Together, these findings support both the robustness and interpretability of the stage-based modeling framework.

## Discussion

In this study, we evaluated the prognostic value of LUS for BPD in very preterm infants using both single–time-point and stage-based prediction strategies. Consistent with our Results, single–time-point models based on early postnatal LUS demonstrated moderate to good discrimination, with OOB AUC values ranging from approximately 0.80–0.85, and the highest performance observed for assessments conducted at postnatal day 14 (AUC 0.853). These findings extend existing evidence that early LUS scores are associated with subsequent BPD developmen t(17, 23–25), while also highlighting the limitations of relying exclusively on a single early assessment.

A key finding of this study is that serial updating of LUS information provided incremental predictive information beyond single-time-point assessment. The three-stage model achieved the highest point-estimate discrimination, with an OOB AUC of 0.874 (95% CI 0.835–0.909) and a Brier score of 0.137 (95% CI 0.121–0.155). Relative to the best-performing single-time-point model at day 14, the three-stage strategy showed an improvement in AUC of 0.021 (95% CI 0.005–0.038). The difference between the two- and three-stage strategies was smaller (ΔAUC 0.009, 95% CI 0.001–0.018), whereas the difference between the three- and four-stage strategies was statistically uncertain (ΔAUC 0.006, 95% CI −0.004 to 0.016). Thus, although the three-stage strategy achieved the highest point estimate, extension to the fourth stage provided no clear additional gain.

Importantly, BPD in this study was defined using the 2018 NICHD revised criteria, which assess disease status and severity at 36 weeks’ postmenstrual age (PMA) (20). Within this diagnostic framework, early imaging is intended to identify infants on disease trajectories that culminate in BPD at the diagnostic time point, rather than to establish an early diagnosis. From a methodological perspective, these findings provide empirical justification for dynamic prediction strategies, in which risk estimates are iteratively updated as longitudinal information accrues (26). The superior performance of the three-stage strategy suggests that incorporating LUS information through the third postnatal week, together with prior predicted risk, captures additional dimensions of disease trajectory that are incompletely represented by earlier assessments alone. The limited incremental gain observed with the four-stage model may reflect diminishing returns as assessments approach the diagnostic window and become increasingly influenced by heterogeneous clinical interventions.

The incremental prognostic value associated with LUS assessments through postnatal day 21 is biologically plausible within the context of evolving postnatal lung development. Modern BPD is characterized by disrupted alveolar and vascular development driven by sustained inflammation and cumulative lung injury (1, 8, 27, 28). Early postnatal LUS primarily reflects initial lung injury and transitional physiology, whereas later assessments may therefore reflect persistent abnormalities associated with impaired lung development, even if they do not directly index specific structural processes such as alveolarization.

Consistent with this concept, LUS scores were significantly higher in infants who later developed BPD at all assessed time points, and in multivariable analyses within the three-stage framework, the contemporaneous LUS score at day 21 retained independent predictive value after accounting for earlier information. These findings align with pathological and clinical evidence indicating that the defining features of modern BPD become increasingly apparent over postnatal weeks as lung development diverges from normal trajectories (1, 27).

Previous studies have established the feasibility of using LUS to predict BPD, including prospective and multicenter investigations demonstrating significant associations between early postnatal LUS scores and subsequent BPD outcomes (17, 23, 24). However, most prior LUS-based work has focused on assessments obtained within the first two postnatal weeks and has mainly evaluated early single time points rather than fully stage-updated longitudinal strategies (25, 29–31). As a result, the incremental value of extending LUS assessment beyond this early window has remained unclear.

By directly comparing strategies that incorporate different temporal structures, the present study extends existing literature by demonstrating that inclusion of LUS information through the third postnatal week, within a stage-based framework, yields measurable improvements in discrimination and risk stratification. This conclusion is supported not only by global performance metrics, but also by the structure of multivariable associations and feature importance analyses, in which prior-stage predicted risk and contemporaneous LUS measurements consistently dominated model contributions, whereas baseline clinical variables did not retain independent associations after adjustment.

The overall BPD incidence in our cohort (64.7%) was higher than pooled estimates reported for comparable gestational-age ranges. This may partly reflect the referral profile of our center: as a provincial-level critical maternal care center and a nationally recognized reproductive medicine center with a provincial key neonatal department, our hospital receives a high volume of preterm and very preterm infants, including a disproportionately high-risk population. In addition, as noted in the Limitations, the high observed BPD incidence may partly reflect the requirement for four completed LUS examinations, which likely enriched the cohort for surviving, hospitalized infants. The use of the 2018 NICHD definition and the single-center design may also contribute. Therefore, the observed incidence may not be representative of all very preterm infants, and discrimination estimates should be interpreted with this prevalence in mind.

From a clinical standpoint, the principal implication of these findings is that the timing and integration of LUS assessments matter. LUS is well suited for longitudinal evaluation in neonatal intensive care because it is bedside, radiation-free, and repeatable, with standardized neonatal scanning protocols available and broader recommendations supporting point-of-care ultrasound use in critically ill patients (32). In the three-stage model, improved statistical performance translated into clinically interpretable risk stratification. When infants were grouped according to tertiles of predicted risk, observed BPD incidence increased from 0.26 in the low-risk group to 0.74 in the medium-risk group and 0.94 in the high-risk group, indicating effective separation of clinically meaningful risk categories. Decision curve analyses further demonstrated that multi-stage models, particularly the three-stage approach, provided greater net benefit across a range of clinically relevant threshold probabilities than default treat-all or treat-none strategies. A day-21 high-risk label would not replace early prevention, but could refine later management, such as stricter oxygen targeting, fluid and nutrition optimization, closer monitoring, earlier follow-up planning, and enrollment in late-phase BPD trials.

### Limitations and future directions

Several limitations should be acknowledged. First, requiring completion of all four serial LUS examinations may have selected for infants stable enough to survive and remain in the NICU through nearly one month of life, plausibly excluding the sickest infants and introducing survivorship/selection bias; this may partly explain the relatively high BPD incidence observed in our cohort. Second, this was a single-center study, and external validation is required to assess generalizability; in addition, LUS remains partly operator dependent and requires structured training and standardized protocols (14, 33–35). Third, airway endoscopy and echocardiography were performed only when clinically indicated and were not systematically included in the analysis; chest radiography was likewise performed only when clinically indicated and was not systematically collected, so LUS could not be directly compared with chest radiography in this cohort; dexamethasone exposure was not classified as peri-extubation vs. post-extubation, and detailed phenotypic and gestational-age-stratified analyses were not performed. These factors could therefore not be fully adjusted for. Fourth, LUS findings such as B-lines and consolidation are not specific to BPD and may overlap with pulmonary edema, pneumonia, or atelectasis, so LUS was interpreted in the clinical context rather than as an etiological diagnostic tool. Finally, BPD was defined by the 2018 NICHD criteria, and later clinical interventions were not fully incorporated into the models; future studies should examine whether the incremental value of LUS through the third postnatal week persists across alternative BPD definitions and severity classifications.

## Conclusions

In summary, early postnatal lung ultrasound provides useful baseline information for BPD risk assessment in very preterm infants, but does not fully capture subsequent disease evolution. Our findings indicate that incorporating LUS information through postnatal day 21 within a time-updated modeling framework yields measurable improvements in discrimination, and clinically meaningful risk stratification compared with reliance on earlier assessments alone. These results underscore the importance of aligning predictive strategies with the temporal progression of neonatal lung disease.

## Data Availability

The raw data supporting the conclusions of this article will be made available by the authors, without undue reservation.
