# Supplementary material for: A stage-based dynamic model for predicting bronchopulmonary dysplasia in very preterm infants using longitudinal lung ultrasound: a retrospective cohort study
Source: Front Pediatr. 2026 Oct 28;14:1928918. doi: 10.3389/fped.2026.1928918 (PMC13752193; doi:10.3389/fped.2026.1928918)
Supplement: Supplementary file 1 [file Supplementaryfile1.docx]

**Supplementary Table S1. Baseline characteristics of infants and mothers**

| Variable | Non-BPD group (n = 95) | BPD group (n = 174) | P value |
| --- | --- | --- | --- |
| Mode of conception (natural / ovulation induction / artificial insemination / IVF) | 55.8% / 4.2% / 32.6% / 7.4% | 57.5% / 36.2% / 4.0% / 2.3% | 0.248 |
| Singleton / twins / triplets | 55.8% / 40.0% / 4.2% | 60.9% / 34.5% / 4.6% | 0.667 |
| Time from last antenatal steroid administration (h) | 16.00 [1, 149] | 12.50 [0, 96] | 0.245 |
| Premature rupture of membranes (h) | 0.00 [0, 0] | 0.00 [0, 6.25] | 0.362 |
| Mode of delivery (vaginal / cesarean / assisted) | 23.2% / 76.8% / 0% | 21.8% / 76.4% / 1.7% | 0.430 |
| Umbilical cord status (normal / abnormal / other) | 81.1% / 11.6% / 7.4% | 86.2% / 9.2% / 4.6% | 0.499 |
| Placental status (normal / abruption / previa / acute chorioamnionitis / other) | 12.6% / 71.6% / 4.2% / 7.4% / 4.2% | 7.5% / 80.5% / 4.6% / 2.9% / 4.6% | 0.266 |
| Hypertensive disorders of pregnancy (none / severe preeclampsia / chronic hypertension with preeclampsia / other) | 87.4% / 5.3% / 3.2% / 4.2% | 74.7% / 10.3% / 8.6% / 6.3% | 0.097 |
| Gestational diabetes mellitus | 5.3% | 3.4% | 0.473 |
| HELLP syndrome | 1.1% | 4.6% | 0.166 |
| Intrahepatic cholestasis of pregnancy | 0% | 1.1% | 0.541 |
| Maternal heart disease | 5.3% | 1.7% | 0.135 |
| Systemic lupus erythematosus (SLE) | 2.1% | 1.1% | 0.616 |
| Other maternal infections | 10.5% | 13.8% | 0.665 |
| Acute chorioamnionitis | 1.1% | 2.3% | 0.659 |
| HIV / syphilis / hepatitis B infection | 0% / 5.3% / 1.1% | 0% / 7.5% / 0% | 0.319 |
| Adequate antenatal steroid exposure | 62.1% | 59.8% | 0.890 |
| Number of steroid doses (0 / 4 / other) | 84.2% / 9.5% / 6.3% | 74.1% / 20.7% / 5.2% | 0.062 |
| Cervical insufficiency | 7.4% | 12.1% | 0.228 |
| Cervical cerclage | 13.7% | 13.8% | 0.980 |
| Hypothyroidism | 4.2% | 4.6% | 1.000 |
| Anemia | 7.4% | 14.8% | 0.017* |

Notes

Abbreviations: **BPD**, bronchopulmonary dysplasia; **IVF**, in vitro fertilization; **HELLP**, hemolysis, elevated liver enzymes, and low platelet count; **SLE**, systemic lupus erythematosus; **HIV**, human immunodeficiency virus.

* P < 0.05 indicates statistical significance.

**Supplementary Table S2. Confusion matrix-derived performance metrics of prediction models**

| Model | Algorithm | Sens | Spec | PPV | NPV |
| --- | --- | --- | --- | --- | --- |
| single_LUS7 | hgbt | 0.80 | 0.75 | 0.85 | 0.68 |
|  | lr | 0.70 | 0.84 | 0.89 | 0.60 |
|  | mlp | 0.75 | 0.77 | 0.86 | 0.63 |
|  | rf | 0.82 | 0.79 | 0.88 | 0.70 |
|  | stacking | 0.84 | 0.76 | 0.86 | 0.72 |
| single_LUS14 | hgbt | 0.77 | 0.79 | 0.87 | 0.65 |
|  | lr | 0.78 | 0.83 | 0.89 | 0.67 |
|  | mlp | 0.71 | 0.83 | 0.89 | 0.61 |
|  | rf | 0.77 | 0.83 | 0.89 | 0.66 |
|  | stacking | 0.76 | 0.84 | 0.90 | 0.66 |
| single_LUS21 | hgbt | 0.72 | 0.76 | 0.85 | 0.60 |
|  | lr | 0.83 | 0.73 | 0.85 | 0.70 |
|  | mlp | 0.78 | 0.75 | 0.85 | 0.65 |
|  | rf | 0.82 | 0.74 | 0.85 | 0.69 |
|  | stacking | 0.69 | 0.86 | 0.90 | 0.60 |
| single_LUS28 | hgbt | 0.83 | 0.71 | 0.84 | 0.69 |
|  | lr | 0.68 | 0.86 | 0.90 | 0.59 |
|  | mlp | 0.67 | 0.84 | 0.89 | 0.58 |
|  | rf | 0.68 | 0.88 | 0.92 | 0.60 |
|  | stacking | 0.71 | 0.86 | 0.90 | 0.62 |
| two_stage | hgbt | 0.82 | 0.79 | 0.88 | 0.71 |
|  | lr | 0.82 | 0.84 | 0.91 | 0.72 |
|  | mlp | 0.89 | 0.66 | 0.83 | 0.76 |
|  | rf | 0.83 | 0.79 | 0.88 | 0.71 |
|  | stacking | 0.77 | 0.86 | 0.91 | 0.67 |
| three_stage | hgbt | 0.80 | 0.83 | 0.90 | 0.69 |
|  | lr | 0.83 | 0.80 | 0.88 | 0.72 |
|  | mlp | 0.75 | 0.84 | 0.90 | 0.65 |
|  | rf | 0.83 | 0.84 | 0.91 | 0.73 |
|  | stacking | 0.82 | 0.85 | 0.91 | 0.72 |
| four_stage | hgbt | 0.78 | 0.85 | 0.91 | 0.68 |
|  | lr | 0.80 | 0.83 | 0.90 | 0.70 |
|  | mlp | 0.75 | 0.81 | 0.88 | 0.64 |
|  | rf | 0.79 | 0.86 | 0.91 | 0.69 |
|  | stacking | 0.81 | 0.84 | 0.90 | 0.71 |

**Abbreviations:**

**BPD**: bronchopulmonary dysplasia; **LUS**: lung ultrasound; **single_LUS7**: single-time-point model based on lung ultrasound data obtained on postnatal day 7; **single_LUS14**: single-time-point model based on lung ultrasound data obtained on postnatal day 14; **single_LUS21**: single-time-point model based on lung ultrasound data obtained on postnatal day 21; **single_LUS28**: single-time-point model based on lung ultrasound data obtained on postnatal day 28; **two_stage**: multi-stage model integrating lung ultrasound data from two early postnatal time points; **three_stage**: multi-stage model integrating lung ultrasound data from three postnatal time points; **four_stage**: multi-stage model integrating lung ultrasound data from four postnatal time points; **Sens**: sensitivity; **Spec**: specificity; **PPV**: positive predictive value; **NPV**: negative predictive value; **LR**: logistic regression; **HGBT**: gradient boosting decision tree; **RF**: random forest; **MLP**: multilayer perceptron; Stacking: stacking ensemble model

**Supplementary Table S3. Robustness analysis under different model specifications (L2-regularized logistic regression)**

| **Model specification** | **No. selected features** | **AUC**  **(OOB)** | **Brier score**  **(OOB)** |
| --- | --- | --- | --- |
| Three-stage | 13 | 0.871 | 0.137 |
| Four-stage | 13 | 0.868 | 0.137 |
| Two-stage | 13 | 0.865 | 0.140 |
| Single time-point model using LUS at postnatal day 14 (single_LUS14) | 12 | 0.859 | 0.148 |
| Single time-point model using LUS at postnatal day 21 (single_LUS21) | 12 | 0.840 | 0.157 |
| Single time-point model using LUS at postnatal day 7 (single_LUS7) | 12 | 0.833 | 0.158 |
| Single time-point model using LUS at postnatal day 28 (single_LUS28) | 12 | 0.826 | 0.161 |

Notes:

Abbreviations:OOB, out-of-bag; OOF, out-of-fold; AUC, area under the receiver operating characteristic curve.

Model specification definitions: Two-stage/Three-stage/Four-stage models incorporate prior-stage OOF risk scores (_prev7/_prev14/_prev21) together with baseline variables and the current LUS time point; Single time-point models use baseline variables plus a single LUS measurement at the specified postnatal day.

**Supplementary Table S4. Feature importance of variables included in the three-stage model across different algorithms**

| **Variable** | **HGBT** | **RF** | **MLP** | **LR** | **Stacking** |
| --- | --- | --- | --- | --- | --- |
| ****_prev14**** | ****0.217**** | ****0.222**** | ****0.153**** | ****0.180**** | ****0.211**** |
| ****LUS21**** | ****0.056**** | ****0.038**** | ****0.106**** | ****0.067**** | ****0.047**** |
| Pre-pregnancy weight | 0.021 | 0.010 | 0.042 | <0.001 | 0.012 |
| Gravidity | 0.006 | 0.003 | 0.036 | <0.001 | 0.007 |
| Parity | 0.004 | 0.002 | 0.025 | 0.001 | 0.004 |
| Maternal age | 0.010 | 0.008 | 0.031 | <0.001 | 0.010 |
| Sex | <0.001 | <0.001 | 0.040 | <0.001 | 0.004 |
| PROM duration | 0.004 | 0.003 | 0.016 | <0.001 | 0.004 |
| Antenatal steroid timing | 0.014 | 0.009 | 0.028 | <0.001 | 0.013 |
| Time to surfactant | 0.013 | 0.012 | 0.024 | ≈0 | 0.015 |
| Apgar score (1 min) | 0.011 | 0.007 | 0.023 | <0.001 | 0.009 |
| Apgar score (5 min) | 0.002 | 0.002 | 0.018 | <0.001 | 0.005 |
| Apgar score (10 min) | <0.001 | <0.001 | 0.009 | <0.001 | 0.003 |

Abbreviations: LUS, lung ultrasound; OOF, out-of-fold; HGBT, histogram-based gradient boosting tree;RF, random forest; MLP, multilayer perceptron; LR, logistic regression.

The three-stage model integrates lung ultrasound (LUS) scores obtained at postnatal days 7, 14, and 21 in a stage-based manner. At each stage, a predictive model is trained using baseline clinical variables and the current LUS measurement. The out-of-fold predicted probability of BPD from the preceding stage (_prev14) is incorporated as an input feature at the subsequent stage.

**Supplementary Table S5. Paired bootstrap comparisons of predictive performance between the three-stage model and alternative staging strategies**

| Comparison | ΔAUC | 95% CI for ΔAUC | ΔBrier score | 95% CI for ΔBrier |
| --- | --- | --- | --- | --- |
| Three-stage vs Single_LUS14 | +0.021 | +0.005 to +0.038 | −0.011 | −0.020 to −0.002 |
| Three-stage vs Two-stage | +0.009 | +0.001 to +0.018 | −0.003 | −0.009 to +0.003 |
| Three-stage vs Four-stage | +0.006 | −0.004 to +0.016 | 0.000 | −0.006 to +0.006 |

Notes:

ΔAUC was calculated as AUC_three-stage − AUC_comparator; positive values therefore indicate higher discrimination for the three-stage model. ΔBrier score was calculated as Brier_three-stage − Brier_comparator; negative values therefore indicate lower prediction error for the three-stage model. Confidence intervals were obtained from paired bootstrap distributions using the same 1,000 resamples.
